# Early-life gut microbiome modulation reduces the abundance of antibiotic-resistant bacteria

**DOI:** 10.1186/s13756-019-0583-6

**Published:** 2019-08-14

**Authors:** Giorgio Casaburi, Rebbeca M. Duar, Daniel P. Vance, Ryan Mitchell, Lindsey Contreras, Steven A. Frese, Jennifer T. Smilowitz, Mark A. Underwood

**Affiliations:** 1 Evolve Biosystems, Inc, Davis, CA 95618 USA; 2 Department of Food Science and Technology, University of Nebraska, Lincoln, NE 68588 USA; 3 Department of Food Science and Technology, University of California, Davis, CA 95616 USA; 4 Foods for Health Institute, University of California, Davis, CA 95616 USA; 5 Department of Pediatrics, UC Davis Children’s Hospital, Sacramento, CA 95817 USA

**Keywords:** Antibiotic resistance gene, Metagenomics, Probiotics, Host-microbe interactions, Microbiology

## Abstract

**Background:**

Antibiotic-resistant (AR) bacteria are a global threat. AR bacteria can be acquired in early life and have long-term sequelae. Limiting the spread of antibiotic resistance without triggering the development of additional resistance mechanisms is of immense clinical value. Here, we show how the infant gut microbiome can be modified, resulting in a significant reduction of AR genes (ARGs) and the potentially pathogenic bacteria that harbor them.

**Methods:**

The gut microbiome was characterized using shotgun metagenomics of fecal samples from two groups of healthy, term breastfed infants. One group was fed *B. infantis* EVC001 in addition to receiving lactation support (*n* = 29, EVC001-fed), while the other received lactation support alone (*n* = 31, controls). Coliforms were isolated from fecal samples and genome sequenced, as well as tested for minimal inhibitory concentrations against clinically relevant antibiotics.

**Results:**

Infants fed *B. infantis* EVC001 exhibited a change to the gut microbiome, resulting in a 90% lower level of ARGs compared to controls. ARGs that differed significantly between groups were predicted to confer resistance to beta lactams, fluoroquinolones, or multiple drug classes, the majority of which belonged to *Escherichia*, *Clostridium,* and *Staphylococcus*. Minimal inhibitory concentration assays confirmed the resistance phenotypes among isolates with these genes. Notably, we found extended-spectrum beta lactamases among healthy, vaginally delivered breastfed infants who had never been exposed to antibiotics.

**Conclusions:**

Colonization of the gut of breastfed infants by a single strain of *B. longum* subsp. *infantis* had a profound impact on the fecal metagenome, including a reduction in ARGs. This highlights the importance of developing novel approaches to limit the spread of these genes among clinically relevant bacteria. Future studies are needed to determine whether colonization with *B. infantis* EVC001 decreases the incidence of AR infections in breastfed infants.

**Trial registration:**

This clinical trial was registered at ClinicalTrials.gov, NCT02457338.

**Electronic supplementary material:**

The online version of this article (10.1186/s13756-019-0583-6) contains supplementary material, which is available to authorized users.

## Background

Each year, more than two million people in the United States develop antibiotic-resistant infections, and at least 23,000 die as a result [1]. Furthermore, it is estimated that over 70% of the bacteria responsible for healthcare associated infections are resistant to at least one of the antibiotics used worldwide as a first-line therapy [2, 3]. Consequently, drug-resistant bacterial infections require longer treatment periods, which cost the US health care system an estimated $5 billion annually [4].

Antibiotic resistance is a natural phenomenon with ancient origins and a product of evolution, occurring in the environment long before the Anthropocene [5]; however, it has accelerated since the commercial development of antibiotics in the twentieth century. In particular, the increased administration of antibiotics to humans and animals has sparked a tremendous selective pressure favoring bacteria that harbor genetic elements that confer antibiotic resistance [6]. Many of these elements are transposable and increase in prevalence as a direct result of human activity mediated by exposure to antibiotics, disinfectants, and heavy metals [7, 8].

The rapid escalation of next-generation sequencing technologies, particularly metagenomics, has produced a remarkable amount of data profiling the ‘resistome’ of environmental niches, with the human gut microbiome itself emerging as a major reservoir for ARGs among commensal organisms [9, 10]. Collectively, the human gut microbiome harbors more ARGs compared to other environments [6, 10–12]. Furthermore, commensal bacteria likely play a key role in the evolution and dissemination of ARGs, even if they are not the intended target of antibiotic therapies [13, 14], and recent evidence demonstrates that maternal gut microbes harboring ARGs are transferred to newborn infants during or shortly after birth [15, 16]. This represents a substantial risk to human health, as ARGs can be transferred from commensal bacteria obtained at birth to pathogens [17] and both can easily be disseminated between individuals. Moreover, resistance genes can persist for years in the human gut, even without long-term antibiotic exposure. For instance, ARGs have been identified up to four years after antibiotic treatment has ceased, indicating that antibiotic resistance can persist for an indeterminate amount of time in the absence of selective pressure [18].

Historically, *Bifidobacterium* are speculated to have been more abundant in healthy breastfed infants in the US and Europe than they are today, given their differential abundance among US and European infants relative to sub-Saharan Africa and southeast Asia, where traditional birth and infant feeding practices are predominant [19–22]. Nonetheless, when bifidobacteria are absent, other taxa including *Proteobacteriaceae, Bacteroidaceae, Staphylococcaceae, Clostridiaceae* predominate in the breastfed infant gut [23–25]. Although these taxa have been associated with potentially negative long-term health outcomes to varying degrees [26], they are also the primary reservoirs of clinically relevant ARGs [10]. We recently demonstrated that extensive and durable changes occur in the breastfed infant gut microbiome resulting from the colonization of *Bifidobacterium longum* subsp. *infantis* (*B. infantis*) EVC001 [25], while others have demonstrated that breastfed infants with higher levels of bifidobacteria have a reduced abundance and lower frequency of genes associated with antibiotic resistance [24, 27].

Here, we used shotgun metagenomics to characterize the effect of an intervention with *B. infantis* EVC001 on the abundance of ARGs in breastfed infants. We found that colonization by *B. infantis* EVC001 resulted in a significant reduction in ARGs and the bacteria that harbor them.

## Materials and methods

### Sample collection

The details of the clinical trial design have been previously reported [25, 28]. Briefly, mother-infant dyads were recruited in the Davis and Sacramento metropolitan region of Northern California (USA), and informed consent was obtained from the mothers prior to enrollment. Only one subject, in the control group, received antibiotics directly prior to sample collection and sequencing. Otherwise, 29% of control infants and 31% of EVC001-fed infants were born by cesarean section, 32% of control infants and 45% of EVC001-fed infants were born to mothers given antibiotics for labor and 16% of control infants and 31% of EVC001-fed infants were born to mothers who tested as Group B *Streptococcus* positive. None of these clinical variables were significantly different between the groups (Additional file 1: Table S1). Infants in the EVC001-fed group were fed 1.8 × 10^10^ CFU of *B. infantis* EVC001 (ATCC SD-7035) for 21 consecutive days from day 7 to day 27 postnatal. *B. infantis* EVC001 was delivered as 156 mg of live bacteria (> 1.8 × 10^10^ CFU) diluted in 469 mg of lactose as an excipient and packaged in single use sachets. Mothers were trained by lactation consultants to mix the contents of the sachet in 5 mL of expressed breast milk and feed this to the infant each day. The probiotic was stored at − 20 °C by the families during the study and stability at − 20 °C was confirmed by a plate count.

### DNA isolation and shotgun metagenomics sequencing

DNA was previously extracted from approximately 100 mg of frozen stools collected from infants on day 21 postnatal [25]. Briefly, the DNA was subjected to bead beating prior to column purification using a Zymo Fecal DNA Miniprep kit, according to the manufacturer’s instructions. Samples with remaining raw material and sufficient extracted DNA meeting input quality standards, as defined by the manufacturer, were selected for sequencing. Sixty samples meeting these criteria were identified. Metagenomic shotgun sequencing was performed at the California Institute for Quantitative Biosciences (QB3) (University of California, Berkeley) on an Illumina HiSeq 4000 platform using a paired-end sequencing approach with a targeted read length of 150 bp and an insert size of 150 bp.

### Quality filtering and removal of human sequences

Demultiplexed fastq sequences were quality filtered, including adaptor trimming using Trimmomatic v0.36 [29] with default parameters. Quality-filtered sequences were screened to remove human sequences using GenCoF v1.0 [30] against a non-redundant version of the Genome Reference Consortium Human Build 38, patch release 7 (GRCh38_p7; www.ncbi.nlm.nih.gov). Human sequence-filtered raw reads were deposited in the Sequence Read Archive (SRA; https://www.ncbi.nlm.nih.gov/sra) under the reference number, PRJNA390646.

### Taxonomic and strain profiling

Taxonomic profiling of the metagenomic samples was performed using MetaPhlAn2 [31], which uses a library of clade-specific markers to provide panmicrobial (bacterial, archaeal, viral, and eukaryotic) profiling (http://huttenhower.sph.harvard.edu/metaphlan2). Strain characterization was performed using PanPhlan [32]. PanPhlan is used in combination with MetaPhlAn2 to characterize strain-level variants in marker genes for a selected organism. For PanPhlan analysis, the pangenomes from *Bifidobacterium longum* (https://bitbucket.org/CibioCM/panphlan) were used as a reference. Both MetaPhlAn2 and PanPhlan were used with their default settings as described in the updated global profiling of the Human Microbiome Project (2017) [33, 34].

### Absolute quantification of *Enterobacteriaceae* by qRT-PC real-time PCR

Quantification of the total *Enterobacteriaceae* was performed by quantitative real-time PCR using the group-specific 16S rRNA gene primers En-lsu3F (TGCCGTAACTTCGGGAGAAGGCA) and En-lsu3 (TCAAGGCTCAATGTTCAGTGTC) [35]. Each reaction contained 10 μL of 2× Power SYBR Green PCR master mix (Applied Biosystems), 0.4 μm of each primer, and 1 μL of template DNA. Thermal cycling was performed on a QuantStudio 3 Real-Time PCR System and consisted of a denaturation step of 10 min at 95 °C followed by 40 cycles of 15 s at 95 °C and 1 min at 60 °C. Standard curves for absolute quantification were generated using genomic DNA extracted from a pure culture of *E. coli* isolated from the fecal sample of an infant (*E. coli* 7005–1) at concentrations ranging from 10^3^ to 10^7^ CFU/mL.

### Antibiotic resistance gene analysis

Human-filtered reads were first subjected to the Humann2 [36] pipeline (http://huttenhower.sph.harvard.edu/humann2) and subsequently screened for ARGs using the DIAMOND v0.9.10 program [37] to conduct a BLASTX-type search against the Comprehensive Antibiotic Resistance Database (CARD) [38] vAugust 2017. Outputs from DIAMOND were parsed and filtered using custom scripts to collect the top hits for each sample considering positive-hits reads with (i) *E*-value ≤10^− 5^ [39]; (ii) amino acid identity ≥90%; and (iii) alignment length ≥ 25 amino acids [40–42]. Tabular outputs were collapsed into a biom format [43] and taxonomic annotation of reads was assigned according to the CARD database and NCBI GeneBank using custom scripts. The final taxonomic affiliation analysis of the ARGs was performed with a MEtaGenome Analyzer (MEGAN v6.9) [44], which was used to compute and interactively explore the taxonomic content of the data set using the lowest common ancestor (LCA) method on the NCBI Taxonomy phylogenetic Tree of Life (LCA parameters: mini-score 35, top percentage 10%). The portion of sequence types or subtypes identified as ARGs in the total metagenome were defined as the relative abundance, using cross-sample normalization in ppm (one read in one million mapped reads) [42, 45].

### PCR amplification and cloning of antibiotic-resistant genes

Seven differentially abundant ARGs identified to be significantly higher in control infants compared to EVC001-colonized infants (*P* ≤ 0.001; Bonferroni) were selected for detection in samples by PCR and functional screening. Illumina reads annotated within the significant ARGs were retrieved and independently assembled in SPADES v3.11 [46]. The assembled sequences were then cross-checked via BLASTX and BLASTP against the NCBI non-redundant protein database (nr, https://www.ncbi.nlm.nih.gov/protein/). To validate the presence of these ARGs in the fecal DNA, primers specific to the open reading frames of each of the seven differentially abundant ARGs were designed using Primer3 (http://bioinfo.ut.ee/primer3-0.4.0/) (Additional file 2: Table S5). Target genes were then amplified separately from 1 μL of fecal DNA from each of six samples with the highest abundance of ARGs according to the metagenomics analysis. Amplified fragments were separated by agarose-gel electrophoresis, purified using the NucleoSpin® kit (Macherey-Nagel) and sequenced using Sanger technology (DNA Sequencing Facility UC Davis). The percent identity of the obtained nucleotide sequences to the corresponding open reading frame of the assembled ARGs was calculated using global pairwise alignment (Needelman-Wunsch). Sequences were also searched against non-redundant protein and nucleotide databases of the NCBI using BLASTX and BLASTN, respectively, and against protein domain databases (Pfam) and COG databases using RPS-BLAST.

### Minimal inhibitory concentrations

Minimal inhibitory concentrations (MICs) were determined according to the Clinical and Laboratory Standards Institute guidelines for microdilution susceptibility testing [47, 48]. Strains grown in LB broth overnight were adjusted to 1 × 10^6^ CFU/mL and inoculated into Mueller-Hinton Broth containing one of six different clinically-relevant antibiotics (ampicillin, tetracycline, cefotaxime, cefazolin, cefepime, and gentamicin) ranging from 0.5 to 512 μg/mL in 96-well polystyrene microtiter plates. The microtiter plates were incubated for 24 h at 37 °C. The optical density (OD) of each well was measured at 600 nm using an automated microtiter plate reader (BIO-TEK, Synergy HT). The MIC corresponded to the lowest antibiotic concentration at which no growth was detected. All tests were performed in triplicate.

### Whole genome sequencing and assembly of bacterial isolates

Approximately 100 mg of fecal sample from day 21 postnatal (subjects 7005, 7084, 7122, and 7174) were serially diluted onto EMB agar and incubated overnight at 37 °C. Three colonies from each subject that were either dark in color and/or had a green metallic sheen were selected for subsequent analysis. Selected isolates were grown overnight in LB broth overnight at 37 °C. For each strain, a 1 mL aliquot was centrifuged at 10,000×*g* for 5 min and the supernatant was removed. The cell pellet was transferred into DNA/RNA Shield Microbe Lysis tubes (Zymo Research, Irvine CA) and high-molecular weight genomic DNA was extracted using a Quick-DNA Fecal/Soil Microbe Miniprep Kit (Zymo Research, Irvine, CA). DNA was extracted following the manufacturer’s protocol with a mechanical lysis in a FastPrep96 (MP Biomedicals, Santa Ana, CA) for 15 s at 1,800 rpm. gDNA integrity was assessed by gel electrophoresis using a high-molecular weight 1 Kb Extension ladder (Invitrogen, Carlsbad, CA). The presence of a gDNA band at 40 kb and no shearing revealed intact gDNA. The gDNA was quantified using a Quant-iT™ dsDNA Assay Kit, high sensitivity (Invitrogen). gDNA purity was assessed using the Take3 microwell UV-Vis system (BioTek, Winooski, VT). Individually barcoded libraries were prepared for each isolate using 400 ng of high-molecular weight gDNA with an Oxford Nanopore 1D Rapid Barcoding Kit (SQK-RBK004) (Oxford Nanopore Technologies, Oxford UK) according to the manufacturer’s protocol. Barcoded samples were pooled and a 1× HighPrep PCR bead clean-up (MagBio, Gaithersburg, MD) of the fragmented and barcoded libraries prior to Rapid adapter ligation was included at the recommendation of Oxford Nanopore. The final 12-plexed pool was loaded onto an R9.4 flow cell and run for 15 h. A secondary run was performed using the same protocol for the seven isolates whose initial coverage was below 6×. Reads were basecalled in real time using MinKnow (ONT, Oxford UK). Data for both runs were combined for subsequent processing. Basecalled reads were demultiplexed and adapters were trimmed using Porechop (version 0.2.3, https://github.com/rrwick/Porechop). Reads were assembled with Canu v1.5 [49] with default parameters. Assembled genomes were converted into local blast databases and the CARD database protein sequences were used as a query against the assembled genomes using TBLASTN with a min *E-*value set at 0.001. Genome assemblies were deposited into the NCBI Gene Bank (https://www.ncbi.nlm.nih.gov/genbank/) under accession number PRJNA472982.

### Statistical analysis

A Mann-Whitney test was used for statistical comparisons between the two groups. Significantly different ARGs between the EVC001-fed infants and controls were estimated using a Kruskal-Wallis one-way analysis of variance, coupled with a Bonferroni correction for cross-sample normalization. A Fisher’s exact test was used to establish a significant presence/absence of gene families in a strain-level analysis. Rarefaction curves were computed to estimate the distribution of the identified ARGs across samples. A nonparametric two-sample *t*-test was used to compare rarefaction curves using Monte Carlo permutations (*n* = 999). A Bray-Curtis dissimilarity matrix was constructed to estimate the global resistome differences among the samples and visualized via a Principal Coordinate Analysis (PCoA). A Permutational Multivariate Analysis of Variance Using Distance Matrices (adonis) was used to assess global resistome differences between treatments and the effect-size (R^2^) of colonization by EVC001 on the resistome. *P*-values for the PCoA panel was computed using F-tests based on sequential sums of squares from permutations of the raw data. *P*-values throughout the manuscript are represented by asterisks (*, *P* < 0.05; **, *P* < 0.01; ***, *P* < 0.001; ****, *P* < 0.0001).

## Results

### *B. infantis* EVC001 reshapes the gut microbiome in breastfed infants

Using shotgun metagenome sequencing, we characterized the taxonomic and antibiotic resistance profiles within the gut microbiome of 60 healthy, term, breastfed infants in Northern California (USA) at 21 days postnatal. Details of the study design and subject characteristics have been previously reported [25, 28] and a summary of the demographic data of the subjects analyzed here is presented in Additional file 1: Table S1. After quality filtering, Illumina sequencing led to a total of 1.6 billion paired-end (PE) reads, of which approximately 3.6% were discarded as human genome sequence contaminants, resulting in an average of 27 million PE reads per sample (Table 1). High-quality, human-filtered reads were subjected to taxonomic profiling following the updated analysis pipeline of the Human Microbiome Project [34] (September, 2017; see Materials and Methods).

**Table 1:** Overview of recovered metagenomics sequencing results from infants fed EVC001 and controls

Sample ID	Library ID in NCBI-SRA	Number of QF reads	Number of human-filtered reads	Mean read length (bp)	Number of ARGs	Supplementation with *B. infantis* EVC001
7020	EBGC1A	16,664,380	11,687,241	139.0	24,584	No
7022	EBGC1B	20,036,684	20,035,538	138.5	763	Yes
7006	EBGC1C	24,650,234	24,611,425	142.2	3,349	Yes
7064	EBGC1D	34,512,182	34,457,072	139.7	784	Yes
7042	EBGC1E	32,069,448	31,976,757	139.1	42,000	No
7085	EBGC1F	33,282,134	31,969,821	142.6	1,267	Yes
7071	EBGC1G	41,857,404	41,821,388	141.0	8,887	No
7018	EBGC1H	31,102,278	30,888,873	144.5	1,246	No
7053	EBGC1I	28,101,472	21,320,795	144.6	707	Yes
7046	EBGC1J	23,792,868	23,789,357	139.7	8,548	Yes
7029	EBGC1K	20,659,444	20,605,385	138.6	8,785	No
7040	EBGC1L	45,898,112	42,404,911	140.9	15,167	No
7002	EBGC1M	25,523,774	22,978,525	140.9	738	Yes
7070	EBGC1N	26,869,998	26,864,626	142.3	754	Yes
7055	EBGC1O	32,042,934	31,924,507	139.4	3,197	No
7025	EBGC1P	26,127,274	23,991,184	142.0	1,897	Yes
7014	EBGC1Q	20,842,834	15,924,419	139.0	22,159	No
7052	EBGC1R	20,538,332	20,105,645	138.8	46,173	No
7074	EBGC1S	32,128,230	32,127,264	141.3	6,301	Yes
7028	EBGC1T	29,018,642	28,901,182	141.2	41,122	No
7077	EBGC2A	38,565,654	38,524,801	141.8	1,068	Yes
7023	EBGC2B	29,636,288	28,626,060	138.8	4,104	No
7054	EBGC2C	31,704,534	31,703,776	137.0	9,46	Yes
7072	EBGC2D	28,452,700	28,389,141	143.1	2,473	Yes
7004	EBGC2E	21,966,018	21,892,596	140.9	25,796	No
7019	EBGC2F	22,137,906	21,102,023	137.1	50,744	No
7005	EBGC2G	24,739,036	23,824,316	137.5	9,539	No
7094	EBGC2H	23,002,228	22,456,171	136.4	6,009	Yes
7079	EBGC2I	29,178,840	29,166,934	139.2	55,263	Yes
7012	EBGC2J	24,605,428	24,554,918	139.9	643	Yes
7035	EBGC2K	22,606,816	22,593,152	142.5	3,228	Yes
7091	EBGC2L	19,907,888	19,866,462	140.3	437	Yes
7007	EBGC2M	19,783,706	19,219,848	139.1	373	Yes
7058	EBGC2O	19,895,006	17,875,331	139.8	2,664	No
7001	EBGC2P	19,391,072	19,383,270	138.6	328	Yes
7032	EBGC2Q	23,850,530	23,837,344	139.9	1,588	Yes
7021	EBGC2R	84,352,444	81,636,315	140.0	107,776	No
7075	EBGC2S	26,710,272	26,301,057	139.4	27,284	No
7067	EBGC2T	20,460,098	20,370,155	138.5	27,587	No
7086	EBGC3A	28,888,864	27,268,596	138.4	51,264	No
7084	EBGC3B	22,770,012	22,182,351	138.4	32,418	No
7068	EBGC3C	23,591,120	23,415,803	138.0	644	Yes
7080	EBGC3D	27,030,836	27,029,113	138.3	416	Yes
7149	EBGC3E	23,871,178	23,862,487	136.8	19,932	No
7076	EBGC3F	48,936,120	48,548,332	138.8	9,874	Yes
7146	EBGC3G	25,966,270	25,897,222	141.1	1,259	Yes
7140	EBGC3H	21,180,698	21,179,628	138.7	219	Yes
7056	EBGC3I	31,171,324	17,196,539	137.4	42,179	No
7174	EBGC3J	20,530,156	20,518,654	138.0	22,767	No
7130	EBGC3K	23,730,582	20,700,297	138.2	17,993	No
7136	EBGC3L	34,194,988	34,053,298	137.5	2,495	Yes
7142	EBGC3M	24,750,628	24,354,502	141.0	1,577	No
7087	EBGC3N	25,393,930	24,853,636	136.4	1,056	Yes
7016	EBGC3O	27,796,252	27,574,657	136.9	8,877	No
7122	EBGC3P	26,488,780	26,470,036	138.6	2,181	No
7050	EBGC3Q	29,278,320	29,179,951	137.3	12,681	No
7051	EBGC3R	23,552,354	23,542,902	134.9	2,028	No
7123	EBGC3S	31,897,560	31,584,466	137.3	1,801	Yes
7015	EBGC3T	24,645,758	24,512,684	136.2	1,300	No
7062	EBGC3U	23,476,062	23,422,279	140.8	36,751	No

**Fig. 1:**
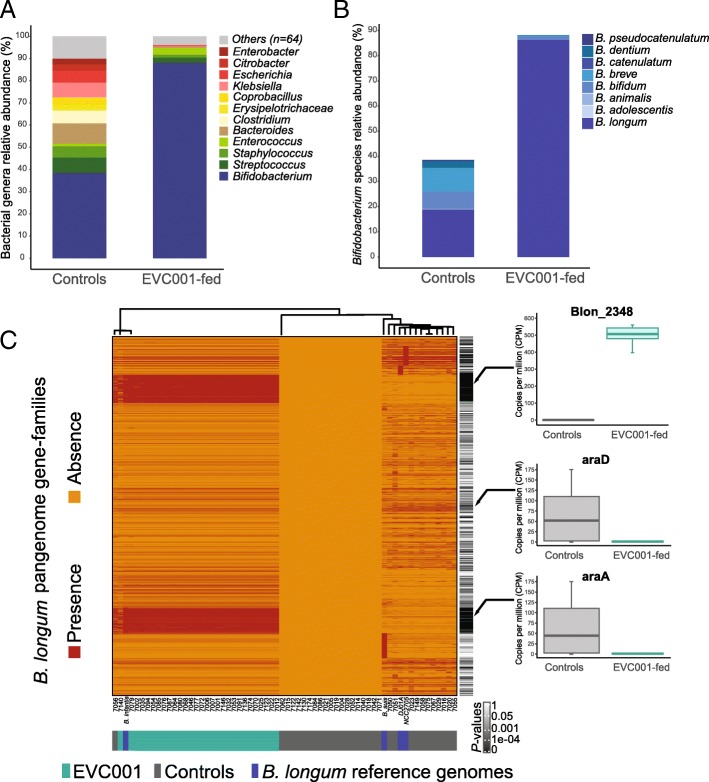
Taxonomic classification of metagenomic reads for EVC001-fed infants and controls. **a** Relative abundance of the top bacterial genera identified between the two groups of infants. **b** Relative abundance of bacterial species belonging to the *Bifidobacterium* genus identified among groups. **c** Hierarchical clustering based on a strain-level analysis of *Bifidobacterium longum* subspecies. Gene family profiles of a subgroup of reference genomes were selected from a global (*n* = 38) strain analysis. Each column represents the presence or absence of genes in a sample or a reference genome with respect to the total pangenome. All samples from EVC001-fed infants clustered together with *B. longum* subsp*. infantis* ATCC 15697 (*B. infantis*), whereas the samples from infants in the control group clustered separately with other *B. longum* subspecies *(*e.g., *B. suis, B. longum* DJ01A*,* and *B. longum* NCC2705). Functional analysis of gene families confirmed that the EVC001 samples were dominated by *B. infantis* due to the presence of unique genes (e.g., Blon_2348 in *B. infantis*), while genes present only in *B. longum* subsp*. longum* (e.g., araD; araA), were abundant in the communities from control infants. *P*-values were computed for each gene via Fisher’s exact test according to group

A total of 202 bacterial species belonging to 76 genera, 43 families, 21 orders, 13 classes, and 7 phyla were identified across the samples (Additional file 3: Table S2). There were differences in the taxonomic distribution between the infants fed EVC001 and those who were not. Among the infants fed EVC001 (*n* = 29), 10 bacterial genera comprised 99% of the community, with the *Bifidobacterium* genus representing 90% of the total relative abundance of any identified genus out of a total of 55 identified genera (*P* < 0.0001, Kruskal-Wallis test) (Fig. 1a).

In the control group (*n* = 31), 68 genera were identified, of which *Bifidobacterium* comprised an average of 38% of the total microbiome, whereas there was a greater proportion of other genera than in the EVC001-fed group, particularly *Clostridium* (*P* = 0.01*,* Kruskal-Wallis test; Fig. 1a)*.* Within the *Bifidobacterium* genus, eight species were identified, of which *Bifidobacterium longum* was the most abundant, representing 86% of the total identified bacterial species within the EVC001-colonized infants and 19% within the controls (*P* < 0.0001, Kruskal-Wallis test; Fig. 1b). Other detected bifidobacteria included *B. breve* and *B. bifidum,* which accounted for 9.4 and 7%, respectively, in the microbiome of the control infants and considerably less (1.4 and 0.4%, respectively) in the EVC001-colonized group.

To discriminate the *B. longum* species at the subspecies level and determine the abundance of *B. longum* subsp. *infantis* and *B. longum* subsp. *longum* to specifically relate changes in the microbiome composition to colonization with *B. infantis*, we performed a strain-level analysis within the *B. longum* species using the pangenome gene-families database provided by PanPhlan. This database includes genes from 38 strains of *B. longum* subspecies (e.g., *B. longum* subsp*. longum, B. longum* subsp*. infantis,* and *B. longum* subsp*. suis*). PanPhlan recovered an average of 98.8% of all genes present in *Bifidobacterium longum* subsp*. infantis* ATCC 15697 [50] from every sample in the EVC001-fed group, representing 2,449 pangenome gene families. In contrast, 19 infants in the control group lacked detectable reads that mapped to *B. longum* subspecies genes in their metagenomes. The remaining control samples (*n* = 12) reported 43% coverage of *B. infantis* genes, and *Bifidobacterium longum* subsp*. longum* NCC2705 displayed the highest gene coverage (79%) across 1,708 pangenome gene families.

Samples and four representative reference genomes were hierarchically clustered based on pair-wise similarities between strains calculated via the Jaccard distance between gene family profiles (Fig. 1c). The resulting heatmap showed that *Bifidobacterium longum* subsp*. infantis* was substantially more abundant than other *Bifidobacterium longum* subspecies in the EVC001 group. Gene loci unique to the *B. infantis* reference genome and samples from *B. infantis* EVC001-fed infants revealed key genes that were more abundant, including human milk oligosaccharide (HMO) utilization clusters [50, 51]. These genes were completely absent among the 29 of 31 infants who were not fed *B. infantis* EVC001, indicating that *B. infantis* was exceptionally rare (only 6% of infants) unless the infants were fed *B. infantis* EVC001. Genes unique to *B. longum* subsp*. longum* that enable characteristic arabinose consumption (araD and araA) were significantly enriched among infants harboring *B. longum* subsp. *longum* and rare among infants colonized by *B. infantis* EVC001. Together, these findings suggest that *B. infantis* was the dominant *B. longum* subspecies among infants fed *B. longum* subsp*. infantis* EVC001, and that *B. infantis* was exceptionally rare among these infants unless the infants were fed the strain during the clinical trial. The high level of *B. infantis* persistence previously reported in infants fed EVC001 in conjunction with the pangenome analysis here suggests stable colonization of the infants by this strain. This is in agreement with other models examining the efficient and durable colonization of host-associated gut microbes in their coevolved host [52, 53] and our prior longitudinal analysis of fecal samples by 16S rDNA sequencing [25]. In terms of microbial diversity, we have previously reported that there was no difference in community richness (alpha diversity) measured via Shannon index, but there are differences in the relative abundance of taxa and community stability (beta diversity) as assessed by UniFrac distance and Jaccard index [25].

### Colonization by EVC001 is associated with a reduced ARG burden

**Fig. 2:**
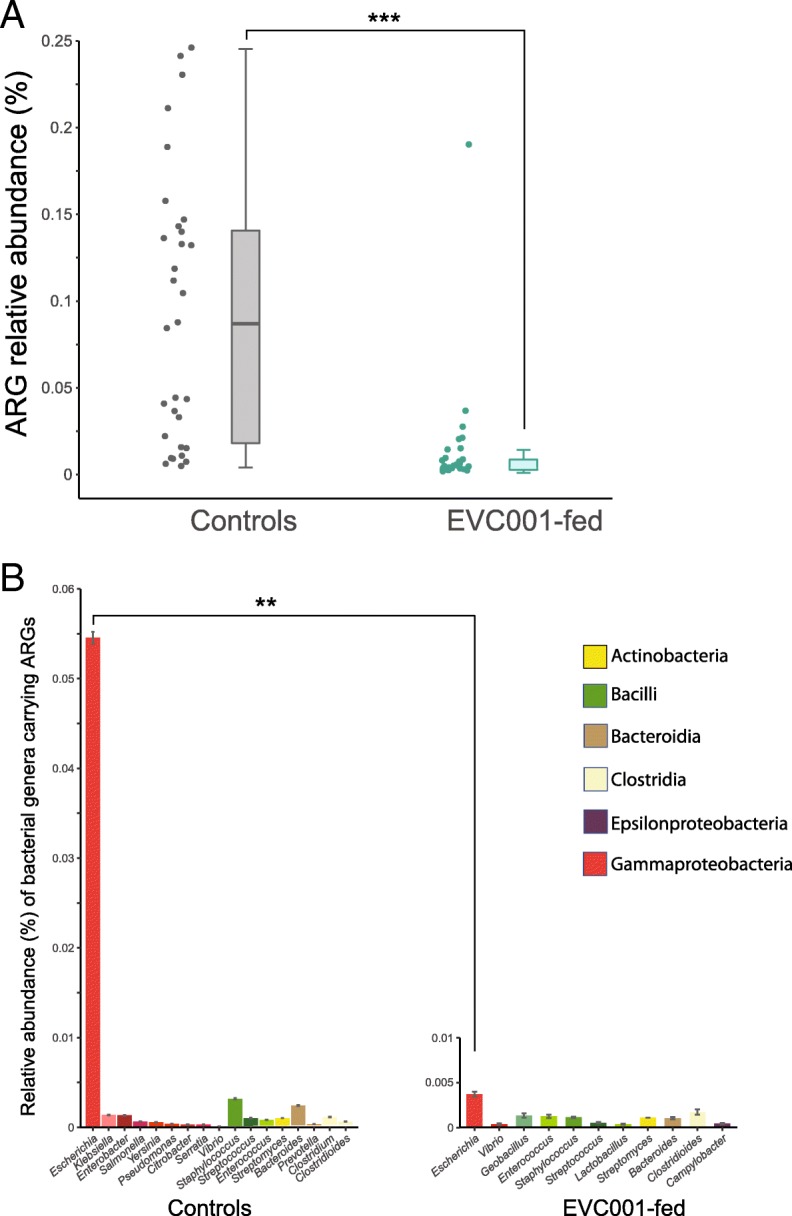
Relative abundance of the total resistome profile in each metagenome sample. **a** Relative abundance of antibiotic resistance genes (ARGs) compared with the overall metagenome for each sample. Each point represents a sample resistome (control, *n* = 31; EVC001-fed, *n* = 29). Box plots denote the interquartile range (IQR), with horizontal lines representing the 25th percentile, median, and 75th percentiles. The whiskers represent the lowest and highest values within 1.5 times the IQR from the first and third quartiles, respectively. The asterisks on the top indicate significant *P*-values (Mann-Whitney test). **b** Relative abundance of ARGs according to their taxonomic identification. The shade of color represents genera belonging to the same bacterial class. The asterisks on the top indicate significant *P*-values (Kruskal-Wallis test)

We identified a total of 599,631 infant gut microbial genes from the shotgun sequencing data, of which 80,925 were unique to 29 infants fed *B. infantis* EVC001, and 313,683 microbial genes were unique to samples from 31 infants not fed *B. infantis* EVC001. Both groups shared a total of 205,023 microbial genes. The metagenomes were screened for ARGs using a BLASTX type search against the curated Comprehensive Antibiotic Resistance Database (CARD). After quality filtering the BLAST results (see Materials and Methods), a total of 652 ARGs were identified (Additional file 4: Table S3). The EVC001-fed group reported an average of 0.01% of ARGs among the total microbial genes (min = 0.001%; max = 0.18%; SEM = 0.006%), with 285 different ARGs (Fig. 2, a), of which 33 were found only in the EVC001 group at very low percentages (< 0.05%). Among the infants not fed *B. infantis* EVC001, these ARGs accounted, on average, for 0.09% of the total metagenomic reads (min = 0.004%; max = 0.24%; SEM = 0.01) with 612 of the different ARGs that were identified. Of these 612 ARGs, 360 uniquely belonged to this group. Thus, infants fed EVC001 had, on average, 90% fewer ARGs in their microbiome (*P* < 0.0001, Mann-Whitney test).

To compare the microbial taxonomic affiliation of ARGs, the 652 ARGs identified among the optimal BLAST hits were assigned to different taxa according to the NCBI taxonomy guidelines coupled with the Lowest Common Ancestor (LCA) method in MEGAN [44]. A total of 41 bacterial genera were taxonomically assigned to the 652 ARGs, of which *Escherichia*, *Staphylococcus, Bacteroides,* and *Clostridioides* were associated with the majority of the ARGs (68.9, 5, 4, and 2.6%, respectively). Considering the taxonomic content within the resistome, the metagenomes from control infants had 17 bacterial genera with a relative abundance > 0.001%, with *Escherichia*-ARGs accounting for about 0.054% of the total metagenome (Fig. 2b). In the EVC001-fed group, only 12 bacterial genera had a relative abundance of associated ARGs > 0.001%. *Escherichia* was also the genus that carried the majority of ARGs but contributed significantly less to the overall metagenome (0.003%) compared with the controls (*P* = 0.001, Kruskal-Wallis test; Fig. 2b).

### EVC001 significantly decreases the abundance of key antibiotic-resistant genes

Among the ARGs uniquely identified in the samples from infants in the control group, three were present in a relative abundance greater than 0.1% and were associated with the *Clostridium* genus. Specifically, tetA(P) and tetB(P), which are ARGs found on the same operon, were identified. tetA(P) is an inner membrane tetracycline efflux protein and tetB(P) is a ribosomal protection protein, both of which confer resistance to tetracycline [54, 55]. mprF was found only in the samples from infants in the control group, and acts by negatively charging phosphatidylglycerol on the bacterial membrane and confers resistance to antibiotic cationic peptides that disrupt the cell membrane, including host-produced defensins [56].

**Fig. 3:**
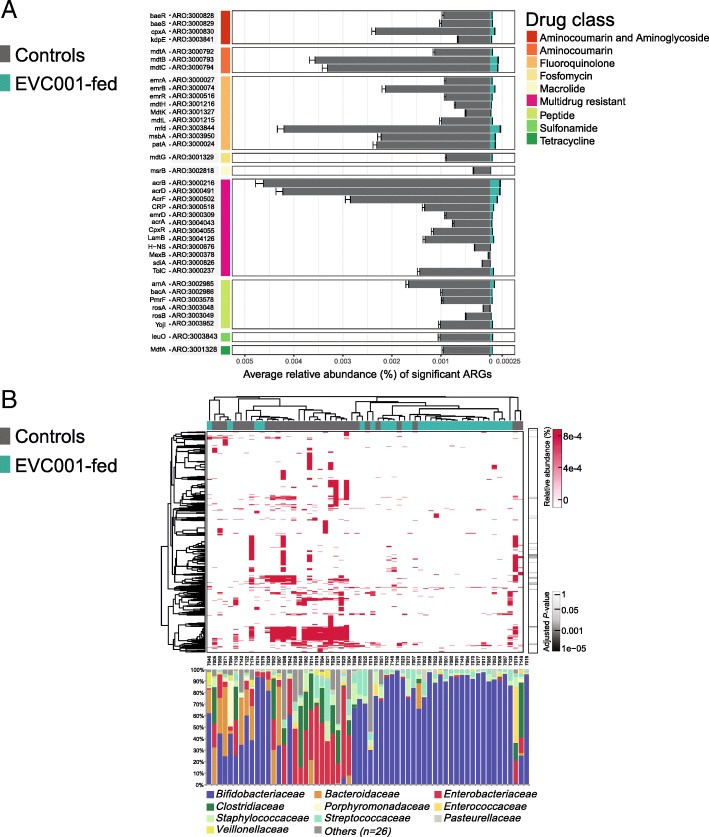
Comparison of the most significant antibiotic resistance gene types. **a** Relative abundance of the most significantly different antibiotic resistance genes (ARGs) identified among EVC001-fed infants and controls (*P* < 0.02; Bonferroni). Percentages are relative to the overall metagenome. These ARGs confer resistance to different drug classes, including beta-lactams, fluoroquinolones, and macrolides. The ARGs are grouped by gene name, followed by CARD identification entry (ARO). The colored bars represent respective drug class to which the ARG is known to confer resistance to. **b** Heatmap showing a hierarchical cluster analysis of the total ARGs identified (*n* = 652). Two groups were identified, one characterized by a lower-ARG carriage, containing most of the samples from infants fed EVC001 and one characterized by a higher overall carriage, containing most samples from infants in the control group. Genes clustered based on similar biological mechanisms implicated in drug resistance (see Results). *P*-values on the bar were computed using a Kruskal-Wallis test normalized with a Bonferroni correction

After cross-sample normalization, 38 ARGs were found to significantly differ between the two groups (*P* < 0.01, Kruskal-Wallis test). All 38 ARGs were significantly lower in the EVC001-fed group compared to the controls (Fig. 3a). Notably, none of the ARGs were significantly higher in the samples from the EVC001-fed group compared to the control group (*P* > 0.05, Kruskal-Wallis test). Genes enriched in the metagenome of infants in the control group were found to confer resistance to beta-lactams, fluoroquinolones, and macrolides, and 12 genes conferred resistance to multiple drug classes.

Hierarchical clustering of the samples and total ARGs (*n* = 652) using the complete-linkage method generated two main clusters of samples (Fig. 3b). The majority of the samples from the EVC001-fed infants were clustered together within the lower-ARGs abundance panel. Row clustering by ARGs resulted in two groups. The most abundant genes clustered together and were annotated as being directly related to mechanisms of antimicrobial resistance. In particular, the proteins encoded by mdtB and mdtC form a heteromultimer complex that results in a multidrug transporter [57]. AcrD is an aminoglycoside efflux pump and its expression is regulated by baeR and cpxAR, which were also identified among the significant ARGs and best characterized in *E. coli* [58]. We also identified AcrB and TolC, which form the multidrug efflux complex, AcrA-AcrB-TolC, that confers multidrug resistance [59]. Moreover, RosA and RosB were significantly more abundant among infants not fed EVC001; these genes form an efflux pump/potassium antiporter system (RosAB) [60]. Three genes belonging to the multidrug efflux system, EmrA-EmrB-TolC, first identified in *E. coli* [61], were also significantly more abundant in the samples from control infants. In this complex, EmrB is an electrochemical-gradient powered transporter, whereas EmrA is the linker, and TolC is the outer membrane channel [62]. The complex confers resistance to fluoroquinolones, nalidixic acid, and thiolactomycin.

**Fig. 4:**
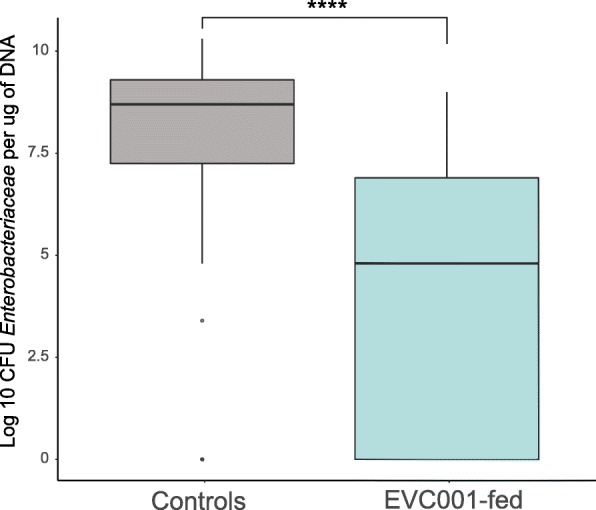
Quantification of *Enterobacteriaceae* family by group-specific qPCR. The data are represented as Log_10_ CFU per μg of genomic DNA extracted from stool samples. Data in boxplots show the median, first, and third quartiles (*P* < 0.0001, Mann-Whitney Test)

The majority (76%) of the significantly different ARGs were taxonomically assigned to bacteria belonging to the *Enterobacteriaceae* family (e.g., *Escherichia coli*) and its abundance was proportional to the presence of ARGs (R = 0.58; *P* < 0.00001, Pearson) (Fig. 3b). The absolute abundance (determined by qPCR) of *Enterobacteriaceae* was significantly lower (*P* < 0.0001) in EVC001-colonized infants (Fig. 4). Other ARGs reported multiple taxonomic assignments within the *Proteobacteria* phylum. According to NCBI’s taxonomic assignment and the CARD database, they could originate from any one of multiple, closely related species. These included: the efflux pump acrD; the MdtG protein, which appears to be a member of the major facilitator superfamily of transporters, that confers resistance to fosfomycin and deoxycholate [63]; BaeR, a response-regulator conferring multidrug resistance [64]; and marA, a global activator protein overexpressed in the presence of different antibiotic classes [65]. A global statistical analysis of ARGs by treatment group is reported in Additional file 5: Table S6.

### Colonization by EVC001 decreases the total abundance and composition of ARGs

**Fig. 5:**
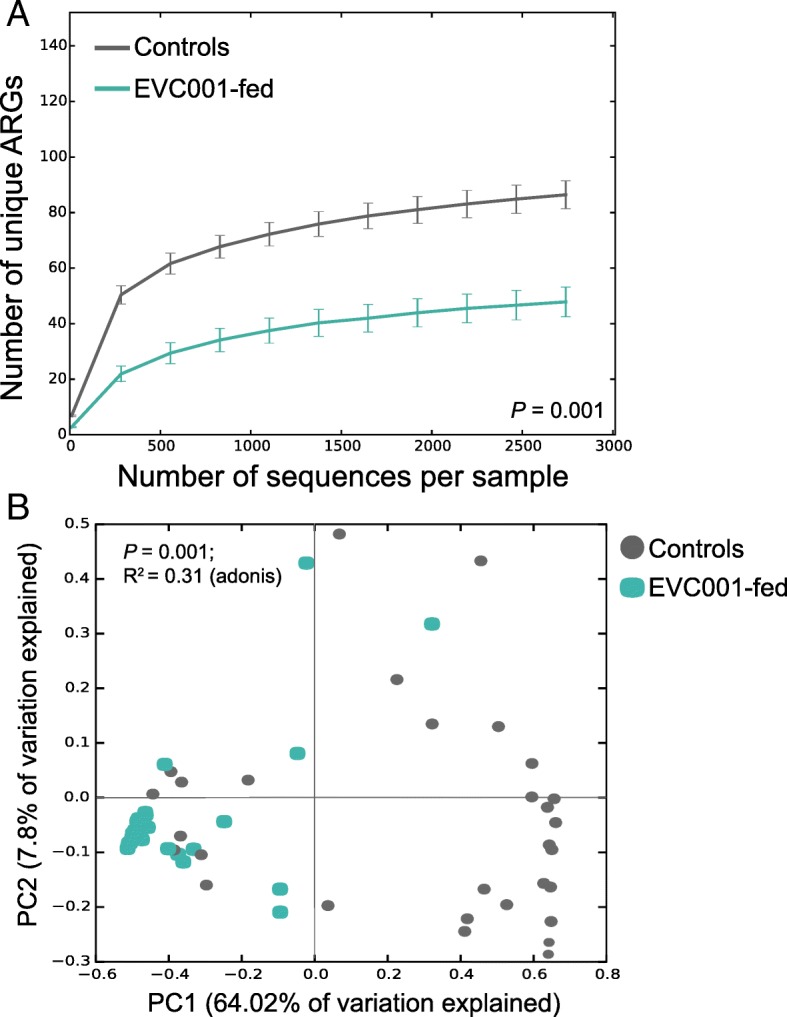
Diversity analyses of infant resistomes according to *B. infantis* EVC001 colonization. **a** Rarefaction curves showing the number of unique antibiotic resistance genes (ARGs) identified in relation to the increasing number of sequences. Both EVC001 and the control group presented similar curve trends, suggesting that the sequencing depth is not associated with the diversity of antibiotic resistance. *P*-values were computed with a nonparametric two-sample *t*-test using Monte Carlo permutations (*n* = 999). **b** Global resistome profiles computed via a principal coordinate analysis (PCoA) based on a Bray-Curtis dissimilarity matrix. The EVC001-fed samples clustered closely, indicating that they shared a similar resistome compared to the controls, which had a more dispersed distribution. The effect of *B. infantis* EVC001 colonization by itself accounted for 31% of the total explained variation (adonis). The *P*-value was computed using F-tests based on the sequential sums of squares from permutations of the raw data

To compare the overall impact of EVC001 colonization on the diversity of ARGs, the alpha-diversity (e.g., number of unique ARGs) within each sample was compared using rarefaction curves. Notably, the diversity of the ARGs was independent from the number of sequences per sample (Fig. 5a). Overall, the EVC001-fed infants had half as many unique ARGs as the control infants (*P* = 0.001, *t-*test). The global resistome differences among samples and the effect-size of EVC001 colonization on the overall diversity of the two study groups were assessed. A Bray-Curtis dissimilarity matrix transposed into a principal coordinate analysis (PCoA) showed that samples from the EVC001-colonized group were clustered closely together compared to the control, which had a wider distribution (Fig. 5b; *P* = 0.001, F-test). This indicates that samples from the EVC001-colonized infants had a less abundant and less diverse resistome compared with the control group samples. Colonization with EVC001 contributed to a greater than a 30% reduction in global AR diversity in the infant gut microbiome than in the gut of the controls (R^2^ = 0.31, *P* = 0.001, adonis). Finally, there was no statistically significant difference in the abundance of ARGs detected in the control group, whether babies were born via C-section or vaginally (*P* = 0.30; Mann–Whitney) or whether infants were exposed to intrapartum antibiotics (*P* = 0.5; Mann–Whitney). Conversely, infants fed *B. infantis* EVC001 had significantly lower ARG abundance, independently from delivery mode, antibiotic exposure or any other clinical variable.

### In vitro validation of in silico predicted ARGs

To validate the ARG presence in fecal samples, PCR primer pairs were designed to target seven of the most abundant ARGs in the resistome of the control infants. Amplicons were obtained in at least half of the analyzed fecal samples, with the exception of the primer pair targeting the mfd gene, which did not amplify from any sample. A nucleotide sequence analysis of the generated amplicons confirmed the sequence identity, with a nucleotide identity of > 70% to the open reading frame (ORF) of the predicted target gene. The nucleotide sequence analysis revealed a high homology (85–99%) with the genomic regions annotated to encode the expected functions in gut bacteria, and the predicted amino acid sequences contained highly conserved structural and functional domains in the corresponding encoded proteins (Additional file 6: Table S4).

To confirm the presence of full length, functional ARGs and the relationship of these ARGs to individual resistance phenotypes at the strain level, bacteria isolated from EMB agar were obtained from the fecal samples of four representative control infants. EMB was used to select for lactose-fermenting coliforms (e.g., *E. coli*) which had been identified by shotgun metagenome sequencing to harbor the most ARGs across individuals in both groups. Whole-genome sequencing of 12 isolates was performed on a MinION sequencer and assembly led to an average coverage of 18× (min 5.4; max 40) (Table 2). Taxonomic identification was confirmed via BLASTN against the NCBI nucleotide database (https://github.com/rrwick/Porechop), revealing three isolates classified as *Raoultella planticola* and the remaining nine as *Escherichia coli.* CARD protein sequence collection was used as a query against the 12 assembled isolates via TBLASTN. The presence of the 40 most differentially abundant ARGs identified via shotgun metagenomics (*P* < 0.02; Bonferroni) was confirmed in all or some of the 12 genomes (average identity > 93%), except for rosB (ARO:3003049), and rob (ARO:3004108). The latter genes were absent from the *E. coli* and *R. planticola* genomes but were predicted in other taxa not isolated here (Table 2).

**Table 2:** Summary of assembly statistics and ARGs assignments for 12 bacterial isolates obtained from four control subjects

		7005–11	7005–5	7005–9	7084–1	7084–4	7084–5	7122–1	7122–2	7122–9	7174–2	7174–4	7174–5
CARD ID	Name	Percentage identity (%) to CARD protein sequences (TBLASTN; *E*-value ≤0.001)											
ARO:3000216	acrB	97.6	97.3	97.8	84.1	84.3	83.1	97.9	96.6	97.8	96.9	95.7	98.0
ARO:3003844	mfd	97.5	96.8	97.0	80.5	80.5	N/A	97.4	N/A	97.3	N/A	95.9	96.4
ARO:3000491	acrD	97.1	97.2	95.2	79.3	79.4	78.2	97.4	96.4	97.4	97.2	97.3	97.4
ARO:3000793	mdtB	95.4	93.8	N/A	81.4	81.4	N/A	95.8	93.3	95.8	91.4	N/A	96.0
ARO:3000794	mdtC	93.5	90.4	N/A	80.8	80.7	N/A	93.2	91.5	93.1	85.3	88.4	93.4
ARO:3000502	AcrF	95.7	93.2	95.6	N/A	N/A	N/A	95.7	94.6	94.9	94.0	91.8	96.4
ARO:3000830	cpxA	97.5	95.3	96.9	N/A	N/A	N/A	96.6	95.2	97.2	96.2	97.0	97.5
ARO:3000024	patA	96.2	95.2	95.4	84.9	84.7	N/A	96.6	96.2	96.7	92.1	96.4	96.7
ARO:3003950	msbA	97.6	96.5	97.3	N/A	N/A	N/A	97.5	N/A	97.5	97.1	94.2	97.5
ARO:3000074	emrB	97.9	97.0	96.0	84.4	84.3	83.6	97.7	96.8	97.6	N/A	95.7	97.9
ARO:3002985	arnA	96.7	96.3	95.4	N/A	N/A	N/A	97.0	96.7	96.9	N/A	95.1	97.0
ARO:3000237	TolC	97.2	96.4	95.3	N/A	N/A	N/A	97.4	97.0	97.5	97.0	91.1	97.2
ARO:3000518	CRP	98.3	98.0	96.1	85.8	85.8	N/A	98.1	95.8	98.1	97.5	98.1	98.3
ARO:3004126	LamB	97.4	96.9	96.5	83.5	83.5	83.6	97.5	97.0	97.8	97.1	97.2	97.5
ARO:3004055	CpxR	98.1	97.3	98.6	84.0	83.7	83.2	98.3	96.9	98.3	97.6	97.4	98.1
ARO:3000792	mdtA	94.6	90.5	N/A	77.5	77.7	N/A	94.8	91.4	95.0	91.0	N/A	95.7
ARO:3002986	bacA	97.2	96.8	95.6	80.7	80.7	79.3	97.7	97.8	97.9	96.4	96.6	97.8
ARO:3003578	PmrF	97.1	96.7	96.7	N/A	N/A	N/A	97.0	96.8	97.3	N/A	95.8	97.2
ARO:3001215	mdtL	95.8	95.6	95.2	N/A	N/A	N/A	96.3	95.5	96.2	95.2	95.8	96.4
ARO:3000027	emrA	96.6	96.4	95.5	N/A	N/A	N/A	97.2	95.4	97.2	92.6	95.8	96.5
ARO:3001328	MdfA	95.5	94.7	N/A	78.1	78.4	N/A	95.5	94.3	95.9	N/A	94.6	95.7
ARO:3003843	leuO	97.4	N/A	95.3	N/A	N/A	N/A	97.5	97.4	97.6	96.8	96.9	97.0
ARO:3003952	YojI	96.2	N/A	96.0	82.6	82.9	78.4	96.0	95.6	96.4	N/A	95.5	95.7
ARO:3001329	mdtG	96.6	95.3	96.5	N/A	N/A	N/A	96.9	93.9	96.9	N/A	96.1	97.2
ARO:3000516	emrR	97.7	97.7	96.8	83.3	N/A	N/A	97.7	96.2	97.7	89.6	96.1	97.7
ARO:3000309	emrD	97.3	96.9	96.8	N/A	N/A	N/A	97.1	97.1	97.4	93.4	95.5	97.1
ARO:3000828	baeR	96.0	94.2	N/A	N/A	N/A	N/A	96.5	94.1	96.4	95.4	95.6	95.8
ARO:3004043	acrA	98.7	97.3	98.0	78.1	78.2	N/A	98.7	97.9	98.5	96.8	90.6	98.7
ARO:3003841	kdpE	95.4	94.9	94.2	N/A	N/A	N/A	95.8	N/A	95.8	93.9	95.8	96.0
ARO:3001216	mdtH	97.9	95.2	97.3	N/A	N/A	N/A	98.0	96.2	98.2	N/A	N/A	97.9
ARO:3003049	rosB	N/A	N/A	N/A	N/A	N/A	N/A	N/A	N/A	N/A	N/A	N/A	N/A
ARO:3004108	rob	N/A	N/A	N/A	N/A	N/A	N/A	N/A	N/A	N/A	N/A	N/A	N/A
ARO:3001327	MdtK	75.1	73.9	74.9	N/A	N/A	N/A	74.8	N/A	74.7	74.4	73.9	75.0
ARO:3000263	marA	96.1	96.1	N/A	N/A	N/A	N/A	96.4	95.1	96.6	96.4	N/A	97.4
ARO:3002818	msrB	98.1	N/A	98.3	89.3	90.4	82.5	97.1	96.2	96.6	N/A	96.6	98.3
ARO:3000676	H-NS	98.1	98.6	95.9	87.1	85.4	83.2	97.3	95.9	98.1	N/A	96.7	98.8
ARO:3003048	rosA	N/A	N/A	N/A	73.9	74.5	72.1	N/A	N/A	N/A	N/A	N/A	N/A
ARO:3000378	MexB	73.2	80	92.3	94.6	83.3	94.7	81.2	91.1	79.1	86.4	100	78.5
ARO:3000826	sdiA	77.4	63.7	N/A	100	81.1	N/A	82.1	85.7	63.1	57.9	N/A	84.2
ARO:3000829	baeS	96.2	58.15	45.0	92.72	70.83	45.7	97.0	53.9	97.6	67.8	93.5	95.2
N. of total CARD hits		75	70	81	38	38	18	74	61	77	226	55	408
Taxonomy		*Escherichia coli*	*Escherichia coli*	*Escherichia coli*	*Raoultella planticola*	*Raoultella planticola*	*Raoultella planticola*	*Escherichia coli*	*Escherichia coli*	*Escherichia coli*	*Escherichia coli*	*Escherichia coli*	*Escherichia coli*
Ref. Genome Size (Mb)		4.8	4.8	4.8	5.8	5.8	5.8	4.8	4.8	4.8	4.8	4.8	4.8
Coverage (based on reference)		22.1x	8.7x	7.5x	14.6x	40x	5.4x	32.7x	8.14x	37.3x	6x	7x	28.6x
Number of contigs		5	53	46	3	1	92	3	57	3	80	66	3
N50 (Mb)		1.57	1.29	1.68	2.32	5.47	4.12	4.67	1.14	4.67	0.51	0.83	4.83

The minimum inhibitory concentration (MIC) to ampicillin, cefepime, cefotaxime, cefazolin, tetracycline, and gentamicin was determined for these isolates. With the exception of three isolates obtained from the same infant (7174), all of the isolates exhibited ampicillin resistance. Among the multidrug-resistance isolates, resistance to ampicillin, cefazolin, and tetracycline was the most common. No resistance to gentamicin was detected (Table 3).

**Table 3:** Minimum inhibitory concentrations (MIC) in μg/mL of antibiotics

Isolate	Ampicillin	Cefepime	Cefotaxime	Cefazolin	Tetracycline	Gentamicin
7005–5	128 (R)	16 (R)	32 (R)	64 (R)	ND	< 4 (S)
7005–9	128 (R)	64 (R)	128(R)	64 (R)	ND	< 4 (S)
7005–11	64 (R)	512 (R)	512 (R)	64 (R)	ND	< 4 (S)
7005–13	64 (R)	512 (R)	512 (R)	64 (R)	ND	< 4 (S)
7084–1	512 (R)	< 4 (S)	< 4 (S)	32 (R)	16 (R)	< 4 (S)
7084–4	512 (R)	< 4 (S)	< 4 (S)	32 (R)	16 (R)	< 4 (S)
7084–5	512 (R)	< 4 (S)	< 4 (S)	32 (R)	16 (R)	< 4 (S)
7122–1	16 (S)	< 4 (S)	< 4 (S)	8 (R)	16 (R)	< 4 (S)
7122–2	16 (S)	< 4 (S)	< 4 (S)	8 (R)	8 (S)	< 4 (S)
7122–9	16 (S)	< 4 (S)	< 4 (S)	8 (R)	8 (S)	< 4 (S)
7174–2	512 (R)	< 4 (S)	< 4 (S)	64 (R)	64 (R)	< 4 (S)
7174–4	512 (R)	< 4 (S)	< 4 (S)	512 (R)	128 (R)	< 4 (S)
7174–5	512 (R)	< 4 (S)	< 4 (S)	512 (R)	64 (R)	< 4 (S)
DH5⍺	< 4(S)	< 4 (S)	< 4 (S)	< 4 (S)	< 4 (S)	< 4 (S)

Resistant (R); Susceptible (S) according to breakpoints for clinical resistance [47, 48]. *ND* not determined

## Discussion

*B. infantis* is a well-known organism with a long history of evolutionary adaptation to the breastfed infant gut [50, 51]. We previously demonstrated that *B. infantis* EVC001 was able to remodel the infant gut microbiome and lower the abundance of common gut taxa, particularly genera belonging to the *Proteobacteria* and *Firmicutes* phyla (e.g., *Escherichia* and *Clostridium*) [23, 25]. In the present study, shotgun metagenomics enabled us to confirm the presence of *B. infantis* as the dominant *B. longum* subspecies among EVC001-fed infants using strain-level analytical tools [32]. Subspecies within *Bifidobacterium longum* present substantial differences in their genetic architecture that have a profound impact on carbohydrate utilization phenotypes [50, 51]. In particular, human milk oligosaccharides (HMOs), which account for the third largest component of human milk, are not digested by the infant and in the absence of *B. infantis,* are lost in the stool [25, 66–68]. Several functions of HMOs have been proposed, including immune signaling molecules, as well as a role as prebiotics [69]. Thus, changes in the HMO concentrations in milk might trigger shifts in the microbiome composition with an impact on health outcomes [70].

To confirm that the dominant *B. longum* subspecies in the EVC001-fed infant gut microbiome was indeed *B. longum* subsp*. infantis*, we collected a pangenome database, composed of 12,000 genes from 38 available subspecies of *B. longum*. A hierarchical cluster analysis based on gene presence or absence, showed that EVC001-fed infants clustered with the *B. infantis* reference genome (ATCC 15697), whereas the control group clustered separately with other *B. longum* subspecies. At the gene level, the HMO cluster-1, which is the largest cluster of HMO-utilization genes and is absent among the *B. longum subsp. longum* strains [50], was present only in the EVC001-fed group but was absent from the control group. In contrast, the L-arabinose isomerase (araA) and L-ribulose-5-phosphate 4-epimerase (araD) that confer the genetic capacity to ferment xylose and arabinose are absent in *B. infantis* but present in *B. longum subspecies longum* [50] and were only found in samples from the control group, which were colonized with *Bifidobacterium longum* subsp. *longum*. Notably, 60% of the control group did not have a detectable amount of any *B. longum* subspecies genes in their metagenomes, suggesting that these bacteria are missing from the microbiome of these infants, regardless of whether they were delivered vaginally or by cesarean section. This could reflect the generational loss of key gut bacterial symbionts in the general population as a result of increasing rates of cesarean section, antibiotic use, and formula feeding over the past century [21, 71, 72].

Given that the infant gut microbiota typically has low colonization resistance during the first two years of life, there is ample opportunity for the infant to acquire antibiotic-resistant populations of commensal organisms, including from the hospital environment [71, 73]. Once established, these resistant populations could contribute to the spread of ARGs. Antibiotic-resistant infections place a substantial health and economic burden on the US health care system and population [74], and there is a limited means by which the spread of ARGs, or the organisms that harbor them, can be restricted [75]. Recent studies have shown that the microorganisms transmitted at birth from the mother and the surrounding environment influence the initial colonization of the infant gut microbiome; however, few studies have investigated the ARG burdens in the gut microbiome of infants colonized by nosocomial bacteria. Recently, Taft et al. showed that an abundance of bifidobacteria is associated with a significant reduction in ARGs in Bangladesh [24]. In the present study, we demonstrated that even in the absence of an antibiotic selective pressure, the healthy term breastfed infant gut of a Northern California population harbors a variety of genes encoding resistance to several clinically relevant antibiotic classes. Furthermore, we demonstrate the possibility of an intervention via modulation of the gut microbiome to decrease the abundance of ARGs. Our results suggest that the effects of targeted probiotic intervention can recapitulate the decrease in ARG abundance observed in *Bifidobacterium*-dominated infants from developing nations [24]. In fact, feeding EVC001 resulted in a significant decrease of 90% in the ARG burden (0.01%) relative to the infants who were not fed EVC001 (0.1%). The computed relative abundance of ARGs in our study was lower compared to what has been found in adults (approximately 0.4–1%) [10, 76], but was similar to other studies in infants [77, 78].

After accounting for cross-sample normalization, we could not identify any ARG increase in the samples from infants fed *B. infantis* EVC001; however, 38 ARGs were significantly increased in the control samples relative to the infants fed EVC001. These genes are known to confer resistance to a variety of drug classes, including fluoroquinolones, beta-lactams, macrolides, and tetracycline antibiotics. A total of 12 significant ARGs were classified as multidrug-resistant genes and reported the widest differences between the two groups. Previous targeted or functional studies on healthy infants have found similar ARGs, including those conferring tetracycline resistance (tet), which we only identified in the control infants [79]; and several multidrug ARGs [80], including CRP, emrD, and CpxR (ARO:3004055)*,* which were the most significantly different genes between the two groups (*P* < 0.0001). Similar ARG abundance and identities reported in our study have also been found in the gut microbiome of preterm infants [81].

These results suggest that the colonization of *B. infantis* EVC001 in infants not only remodeled the gut microbiome composition in breastfed infants, but also may provide an immediate translational impact through a reduction in ARG carriage. Diversity analyses indicated that there were significant reductions in the number of unique ARGs in the EVC001-fed group compared to the controls, and this trend was independent of the sequencing depth. This aspect might translate to clinical relevance, since infants whose infections originate within the gut microbiome could be less likely to exhibit resistance to a wider spectrum of drug classes. Further studies are needed to determine the durability of these ARG profiles following cessation of the probiotic.

Although the use of shotgun metagenomics coupled with clinical databases (e.g., CARD) offer a direct means of identifying ARGs on a global, high-throughput scale, short-read sequencing might represent a challenge when linked to the resistance of specific genes that may originate from multiple strains of the same species [82]. Moreover, the computational annotation of ARGs can be difficult to translate into actual drug resistance, with some evidence showing a disconnect between the predicted and actual phenotypes [83]. Additionally, alternative mechanisms independent from genetic variations, could confer drug resistance, including biofilm formation [84–86]. Similarly, the absence of ARGs may not necessarily translate into non-resistant phenotypes. Nevertheless, by sequencing the ORFs of the most abundant ARGs in the fecal DNA of control infants, we confirmed that these genes were present in the analyzed fecal samples. More importantly, homology searches against well-curated sequence databases (e.g., pfam and COG) confirmed that the obtained gene sequences contained functionally conserved domains. Genome sequencing and phenotypic confirmation of antibiotic-resistant phenotypes confirmed the association between the presence of many of these genes and antibiotic resistance using clinically defined breakpoints, highlighting the fact that these genes are present in predicted organisms and demonstrably confer clinically relevant levels of resistance.

Finally, it is important to note that this study is limited to ARGs found in public databases and thus contains only known genes involved in antibiotic resistance. Therefore, the identified ARGs may be underrepresented, as our analysis excluded chromosomal mutations and novel, resistance genes not found within the CARD database. However, the majority of ARGs are associated with *Proteobacteria* and *Firmicutes*, whose populations were significantly reduced in infants fed *B. infantis* EVC001 compared to those who were not*.* Natural, nontransferable antibiotic resistance phenotypes have long been established among bifidobacteria, and recent systematic analyses have differentiated between natural and acquired *Bifidobacterium* resistance phenotypes [87]; however, *B. infantis* lacks any known virulent genes or plasmids [50], and there are currently no studies describing evidence of horizontal gene transfer events.

## Conclusions

Colonization of newborn infants with antibiotic-resistant bacteria represents a major risk to the continued dissemination of ARGs within human populations. Colonization of the gut microbiome with *B. infantis* EVC001 early in life reduces the abundance of these ARGs as well the taxa that harbor and potentially transmit them between individuals. In conjunction with appropriate drug stewardship practices by the medical community, this approach of modulation of the gut microbiome could help reduce the burden and diversity of ARGs and limit their transmission across individuals. Additional research is required to determine whether these changes have clinically relevant benefits, such as changing the prevalence of antibiotic-resistant nosocomial infections.

## Additional files

Additional file 1: **Table S1.** Study population demographics. (DOC 50 kb)

Additional file 2: **Table S5.** Primers sequences used to amplify seven differentially expressed ARGs from the fecal DNA (DOC 30 kb)

Additional file 3: **Table S2.** Global taxonomic profile by sample. Relative abundance (%) of individual taxa identified across samples. Stratification refers to different taxonomic rank (k = kingdom; p = phylum; c = class; o = order; f = family; g = genus; s = species; t = strain). Within the same taxonomic rank (e.g., Family, “f__”) the total relative abundance sum up to 100%. (XLS 427 kb)

Additional file 4: **Table S3.** Global ARG profile by sample. Number of quality filtered hits for every sample against the CARD database. First column reports the annotation in the following order: protein ID based on NCBI reference sequence database; antibiotic resistance gene ID according to the CARD database and protein name. (XLS 312 kb)

Additional file 5: **Table S6.** Kruskal-Wallis test with post-hoc corrections for every ARG identified according to treatment status. (XLSX 51 kb)

Additional file 6: **Table S4.** Nucleotide sequence analysis of ORFs. Global alignment results of selected open reading frames (ORFs) from Illumina reads assembly. BLASTX and BLASTN scores of selected ORFs identified among ARGs are reported. (XLSX 11 kb)

## Data Availability

The sequencing libraries generated in this study are publicly deposited at the NCBI sequence read archive (SRA) under accession number PRJNA390646. Whole-genome assemblies are deposited in NCBI under accession number PRJNA472982.
