# Supplementary material for: Early-life gut microbiome modulation reduces the abundance of antibiotic-resistant bacteria
Source: Antimicrob Resist Infect Control. 2019 Aug 14;8:131. doi: 10.1186/s13756-019-0583-6 (PMC6693174; doi:10.1186/s13756-019-0583-6)
Supplement: Supplementary file 1 — Table S1. Study population demographics. (DOC 50 kb) [file 13756_2019_583_MOESM1_ESM.doc]

**Table S1. Study population demographics^a^**

| **Measure** | **Controls (SD)^b^** | **EVC001-fed (SD)** | ***P-*value^c^** |
| --- | --- | --- | --- |
| Group Size | 31 | 29 |  |
| Home births | 1 | 1 | NS (*P* > 0.999) |
| Hours in labor | 18.73 (23.09) | 10.85 (10.92) | NS (*P* = 0.41) |
| Cesarean Section Births | 9 | 9 | NS (*P* > 0.9999) |
| Mothers received antibiotics for labor | 10 | 13 | NS (*P* = 0.4267) |
| Gestational Age (weeks) | 40.00 (1.14) | 39.45 (1.15) | NS (*P* = 0.40) |
| Males | 16 | 11 | NS (*P* = 0.31) |
| Birth weight (g) | 3572 (630) | 3421 (367) | NS (*P* = 0.70) |
| Discharged weight (g) | 3365 (619) | 3221 (380) | NS (*P* = 0.70) |
| Birth length (cm) | 51 (3) | 50 (2) | NS (*P* = 0.72) |
| Infants received antibiotics prior to discharge | 1 | 0 | NS (*P* = > 0.99) |
| Birth complications (any) | 6 | 0 | *P* = 0.02 |
| Infants consumed formula before discharge | 2 | 0 | NS (*P* = 0.49) |
| Maternal pre-pregnancy BMI | 23.88 (3.23) | 25.53 (3.78) | NS (*P* = 0.40) |
| Pregnancy weight gain (kg) | 15.33 (5.17) | 14.63 (4.78) | NS (*P* =0.72) |
| Group B Streptococcus positive | 5 | 9 | NS (*P* = 0.23) |
| Primiparous | 7 | 9 | NS (*P* = 0.56) |
| Maternal Age (years) | 31.10 (3.46) | 33.66 (4.17) | NS (*P* = 0.09) |

^a^Additional information has been reported previously^22,23^
 ^b^Values are presented as incidences or means with standard deviations in parentheses.

^c^Statistical tests were performed as Fisher’s exact test or multiple *t*-tests with the Holm-Sidak correction as appropriate.
