# Supplementary material for: Early-life gut microbiome modulation reduces the abundance of antibiotic-resistant bacteria
Source: Antimicrob Resist Infect Control. 2019 Aug 14;8:131. doi: 10.1186/s13756-019-0583-6 (PMC6693174; doi:10.1186/s13756-019-0583-6)
Supplement: Supplementary file 2 — Table S5. Primers sequences used to amplify seven differentially expressed ARGs from the fecal DNA (DOC 30 kb) [file 13756_2019_583_MOESM2_ESM.doc]

| Gene | Forward primer | Reverse primer |
| --- | --- | --- |
| *acrA* | 5’ATGAACAAAAACAGAGGGTT 3’ | 5’TTAAGACTTGGACTGTTCAG 3’ |
| *acrD* | 5’ATGGATCGCGGACGCGTGAA 3’ | 5’TTATTCCGGCCTCTCTTTCA 3’ |
| *cpxA* | 5’ATGATAGGAAGTTTAACCGC 3’ | 5’TTAGGTGCGCTTATACAGCG 3’ |
| *crp* | 5’ATGGTGCTTGGCAAACCGCA 3’ | 5’TTAACGCGTACCGTATACGA 3’ |
| *marA* | 5’ATGATGTCCAGACGCAATAA 3’ | 5’TTAACAGCAGTTATTTAGTG 3’ |
| *mfd* | 5’ATGCGTGGCCAGCGCGAAAC 3’ | 5’TTACAGGCCGCCGAAGGTAA 3’ |
| *msrB* | 5’ATGGCGAATAAACCCTCCTC 3’ | 5’TTAATTGAAGCTTAATGAGG 3’ |

**Table S5. Primers sequences used to amplify seven differentially expressed ARGs from the fecal DNA**
